# Bradykinin B1 Receptor Affects Tumor-Associated Macrophage Activity and Glioblastoma Progression

**DOI:** 10.3390/antiox12081533

**Published:** 2023-07-31

**Authors:** Ching-Kai Shen, Bor-Ren Huang, Vichuda Charoensaensuk, Liang-Yo Yang, Cheng-Fang Tsai, Yu-Shu Liu, Dah-Yuu Lu, Wei-Lan Yeh, Chingju Lin

**Affiliations:** 1 Graduate Institute of Biomedical Science, China Medical University, Taichung 40402, Taiwan; b0953295012@gmail.com; 2 School of Medicine, Tzu Chi University, Hualien 97004, Taiwan; 3 Department of Neurosurgery, Taichung Tzu Chi Hospital, Buddhist Tzu Chi Medical Foundation, Taichung 427213, Taiwan; 4 Department of Pharmacology, School of Medicine, China Medical University, Taichung 40402, Taiwandahyuu@mail.cmu.edu.tw (D.-Y.L.); 5 Department of Physiology, School of Medicine, College of Medicine, China Medical University, Taichung 40402, Taiwan; 6 Department of Medical Laboratory Science and Biotechnology, Asia University, Taichung 41354, Taiwan; 7 Department of Photonics and Communication Engineering, Asia University, Taichung 41354, Taiwan; 8 Department of Biochemistry, School of Medicine, China Medical University, Taichung 40402, Taiwan; 9 Institute of New Drug Development, China Medical University, Taichung 40402, Taiwan

**Keywords:** GBM, B1R, cytokine/chemokine, tumor-associated macrophages, endogenous antioxidant

## Abstract

Bradykinin is a small active peptide and is considered an inflammatory mediator in several pathological conditions. Bradykinin exerts its effects by coupling to its receptors, including bradykinin B1 (B1R) and bradykinin B2. B1R has been implicated in the development of various cancers. Our previous study reported that B1R promoted glioblastoma (GBM) development by supporting the migration and invasion of GBM cells. However, the mechanisms underlying the effects of B1R on tumor-associated macrophages (TAMs) and GBM progression remain unknown. Accordingly, to explore the regulatory effects of B1R overexpression (OE) in GBM on tumor-associated immune cells and tumor progression, we constructed a B1R wild-type plasmid and developed a B1R OE model. The results reveal that B1R OE in GBM promoted the expression of ICAM-1 and VCAM-1—cell adhesion molecules—in GBM. Moreover, B1R OE enhanced GBM cell migration ability and monocyte attachment. B1R also regulated the production of the protumorigenic cytokines and chemokines IL-6, IL-8, CXCL11, and CCL5 in GBM, which contributed to tumor progression. We additionally noted that B1R OE in GBM increased the expression of CD68 in TAMs. Furthermore, B1R OE reduced the level of reactive oxygen species in GBM cells by upregulating heme oxygenase-1, an endogenous antioxidant protein, thereby protecting GBM cells from oxidative stress. Notably, B1R OE upregulated the expression of programmed death-ligand 1 in both GBM cells and macrophages, thus providing resistance against T-cell response. B1R OE in GBM also promoted tumor growth and reduced survival rates in an intracranial xenograft mouse model. These results indicate that B1R expression in GBM promotes TAM activity and modulates GBM progression. Therefore, B1R could be an effective target for therapeutic methods in GBM.

## 1. Introduction

Bradykinin, a member of the kinin family, is considered an inflammatory mediator that is involved in a wide range of physiological effects, including inflammatory responses, cardiovascular homeostasis, pain-inducing mechanisms, neurotransmission, and disease progression [1]. Accumulating evidence demonstrates that bradykinin regulates the permeability of the blood–brain barrier (BBB) during ischemia and tissue injury [2,3]. Bradykinin activates bradykinin B1 (B1R) and B2 (B2R) receptors, which are associated with the pathogenesis of several central nervous system (CNS) disorders [4]. B1R expression—but not B2R expression—was reported to be significantly enhanced in inflammatory CNS lesions in mice and humans [5]. Moreover, bradykinin and its receptors regulate neurogenesis and cerebral angiogenesis [6]. B1R is generally expressed in conditions that are highly correlated with cancer, such as tissue injury, cytokine stimulation, and inflammatory insults [7,8].

Accumulating evidence reveals the involvement of bradykinin and its receptors in several pathological processes associated with cancer [9]. A study reported higher B1R and B2R expression levels in tumor tissues than in normal tissues [10]. Another study revealed that selective inhibition of B1R and B2R by antagonists engendered antiproliferative, antimigratory, and anti-inflammatory effects in tumor cells [11]. Several studies have reported that B1R was highly expressed in gastric, breast, prostate, and lung cancer cells [12,13,14,15]. Bradykinin receptors were also observed to be upregulated in rat and human glioma cells [16]. Moreover, a study demonstrated that B1R mediated glioblastoma (GBM) invasion and that it was involved in interactions between GBM and mesenchymal stem cells [17]. In particular, a study observed the expression of bradykinin receptors in glioma cells and noted that such expression was positively correlated with the World Health Organization tumor grade [18]. However, the mechanisms through which B1R affects tumor-associated macrophages (TAMs) and the tumor microenvironment (TME), thus contributing to GBM progression, are unclear and warrant elucidation.

GBM is a primary brain tumor, and it is the most severe and treatment-resistant tumor among all brain and CNS neoplasms [19,20]. The TME of GBM is highly heterogeneous and dynamic and, thus, plays a substantial role in GBM responses to treatment [21]. A complex TME comprises the nervous system, glioma stem cells, gliomas, immune cells such as TAMs, the brain vascular system, extracellular matrix components, and supporting noncellular chemical compartments, including oxygen and pH [22]. Cell–cell interactions between circulating immune cells and the CNS affect a wide range of cellular properties [23]. Crosstalk between tumor cells and TAMs mediates cancer progression [24]. Notably, brain-infiltrating immune cells around a glioma protect the tumor against chemotherapeutic drugs [25]. During GBM progression, macrophages and microglia are recruited and migrate between epithelial cells and the extracellular matrix through diapedesis, a process that is mediated by the interaction between rolling monocytes and the tumor; this process stimulates increased expression of cell adhesion molecules (CAMs) [26]. Consequently, the aforementioned macrophages and microglia are activated, and TAMs adhere firmly to the tumor [27]. α4β1 integrin mediates the rolling of macrophages and microglia on vascular CAM (VCAM)-1—an α4β1 integrin ligand—thus enabling macrophages and microglia to strongly adhere to this ligand [28]. Zheng et al. observed that the absence of VCAM-1 reduced macrophage binding to GBM cells and GBM cell invasiveness [29]. Furthermore, the expression of intercellular CAM (ICAM)-1—a β2 integrin ligand—and VCAM-1 could be augmented through the induction of the inflammatory cytokines tumor necrosis factor-α and interleukin (IL)-1β [30] and reactive oxygen species (ROS) [31]. We recently reported that IL-1β stimulated the expression of VCAM-1 and ICAM-1 and promoted the adhesion of monocytes to human GBM cells [32]. 

TAMs respond to their microenvironment and polarize into either protumoral or antitumoral macrophages. Antitumoral TAMs support immune functions and exert anticancer effects [33], whereas protumoral TAMs support tumor progression [34]. Glioma-associated macrophages can produce tumor-promoting factors, anti-inflammatory cytokines, and vascular-promoting factors, which may cause immune escape and tumor progression [22]. We conducted the present study to elucidate the mechanisms through which B1R regulates TAMs and TME development. We also investigated the effects of B1R overexpression (OE) in GBM cells on GBM progression.

## 2. Materials and Methods

### 2.1. Materials 

Bradykinin, (Lys-des-Arg9)-bradykinin (LDBK), and primary antibodies against vimentin and GAPDH (1:20,000) were purchased from Sigma-Aldrich (St. Louis, MO, USA). The primary antibody against HO-1 (1:4000) was obtained from StressGen Biotechnologies (San Diego, CA, USA). The primary antibodies against B1R (1:1000), ICAM-1 (1:1000), and α-tubulin (1:10,000) were purchased from Santa Cruz Biotechnology (Santa Cruz, CA, USA). The primary antibodies against VCAM-1 (1:4000), PD-L1 (1:1000), β-catenin (1:10,000), and β-actin (1:10,000) were purchased from Abcam (Cambridge, UK).

### 2.2. Cell Culture

The U251 human glioma cells were acquired from JCRB NO. IFO50288, Japan. The U87 human glioma cells and THP-1 human monocyte were purchased from the Bioresource Collection and Research Center (BCRC No. 60360, 60582, and 60430; Taiwan). The U251 and U87 cells were maintained in Minimum Essential Medium (MEM) with 10% FBS and 1% PS. THP-1 was cultured with RPMI-1640 medium containing 10% FBS, 1% PS, and 2-mercaptoethanol. Cells were kept in a humidified incubator containing 5% CO_2_ and 95% air at 37 °C.

### 2.3. Cell Transfection

Cell transfection was performed according to our previous study [35]. Briefly, GBM cells were cultured on a 6-well plate (3 × 10^5^ cells/well) for 24 h. Then, serum-free MEM medium (1 mL/well) containing plasmids pretreated with 1 μL/mL lipofectamine 3000 (LF3000; Invitrogen, Waltham, MA, USA) transfection agent for 5 min was added to the plate. After transfection at 37 °C for 24 h, the LF3000-containing medium was replaced with fresh serum-free MEM medium. The transfection efficiency was more than 60% [36].

### 2.4. Real-Time PCR (qPCR)

Total RNA was extracted using TRIzol reagent (MDBio Inc., Taipei, Taiwan) and quantified using a BioDrop spectrophotometer (Cambridge, UK). The reverse transcription (RT) reaction was performed by converting 2 μg of total RNA into cDNA using oligo (dT) primers. Quantitative real-time PCR amplification was performed using SYBR Green I Master Mix and analyzed with a StepOne Plus Sequence Detector System (Applied Biosystems, Foster City, CA, USA).

### 2.5. Western Blotting

To prepare the whole-cell lysis extracts, cells were lysed on ice with RIPA lysis buffer (50 mM hydroxyethyl piperazineethanesulfonic acid (HEPES) (pH 7.4), 150 mM NaCl, 4 mM ethylenediaminetetraacetic acid (EDTA), 10 mM Na_4_P_2_O_7_, 100 mM NaF, 2 mM Na_3_VO_4_, 1% Triton X-100, 0.25% sodium deoxycholate, 50 mM 4-(2-aminoethyl) benzenesulfonyl fluoride, 50 μg/mL leupeptin, and 20 μg/mL aprotinin) for 30 min. The protein samples were separated using sodium dodecyl sulfate-polyacrylamide (SDS) gel and blotted onto polyvinylidene difluoride (PVDF) membranes (Millipore, Bedford, MA, USA). The membranes were blocked with 5% nonfat milk in tris-buffered saline and Tween 20 (TBST) for 1 h, washed with TBST three times 10 min each, and probed with the primary antibodies at 4 °C overnight. After undergoing TBST washing, the membranes were incubated with peroxidase-conjugates secondary antibodies (Santa Cruz Biotechnology) for 1 h at room temperature. The blots were visualized using enhanced chemiluminescence (ECL) and Kodak X-OMAT LS film (Eastman Kodak, Rochester, NY, USA).

### 2.6. Cell Migration (Wound Healing) Assay

Cells were transfected with B1R wild-type plasmid or empty vector. Then, 1.5 × 10^4^ cells were plated onto the Culture-Insert (Ibidi, München, Germany). The insert was removed after 4 h. Cell migration was observed under a light microscope at 0 and 24 h.

### 2.7. Flow Cytometry Analysis

Cells were transfected with B1R wild-type plasmid or empty vector for 24 h. On the following day, U251 cells were incubated with antibody against PD-L1 conjugated with APC (BioLegend, San Diego, CA, USA). PD-L1 expression was determined using a flow cytometer (ACEA Biosciences, San Diego, CA, USA).

### 2.8. Monocyte Binding Assay

GBM cells were transfected with an empty vector or B1R wild-type plasmid for 24 h. THP-1 cells were labeled with 200 ng/mL BCEFC/AM (Invitrogen, Carlsbad, CA, USA) for 1 h at 37 °C. Then, 3 × 10^5^ BCEFC/AM-labeled THP-1 cells were added to the GBM cells and incubated at 37 °C. After 45 min of incubation, the medium was removed. The cells were washed gently with culture medium twice. The monocyte-binding ability was then observed under a fluorescence microscope.

### 2.9. ROS Production Analysis

The production of ROS was assessed according to the oxidation of specific probes 2′,7′-dichlorodihydrofluorescein diacetate (DCFDA). U251 cells were plated onto a 3.5 cm dish and transfected with B1R wild-type plasmid or empty vector for 24 h. The cells were treated with DCFDA (10 μM) for 40 min at 37 °C. The fluorescence intensity was measured with an excitation filter of 525 nm emission wavelengths using a flow cytometer.

### 2.10. Co-Culture of GBM Cells and Macrophages

U251-GFP cells were transfected with B1R wild-type plasmid or empty vector for 24 h and co-cultured with human THP-1 differentiated macrophage (HM) for an additional 24 h. U251 cells were incubated with anti-CD68-conjugated PE (Santa Cruz), anti-CD206-conjugated PE (eBioscience, San Diego, CA, USA), and anti-CD163-conjugated APC (BioLegend) antibodies. The expression of these proteins was determined using a flow cytometer.

### 2.11. Animals

Ten-week-old male C57BL/6 mice were obtained from the National Laboratory Animal Center (Taipei, Taiwan). The mice were kept under standard laboratory conditions (21 ± 2 °C, 12 h light/dark cycle) with food and water available ad libitum. All animal procedures were carried out in accordance with the Institutional Animal Care and Use Committee (IACUC) of China Medical University (Taichung, Taiwan). 

### 2.12. Intracranial Tumor Models

Mouse ALTS1C1 cells were transfected with an empty vector or B1R wild-type plasmid for 48 h. Empty vector-transfected ALTS1C1 (3 × 10^4^ cells) or B1R-overexpressing ALTS1C1 cells (3 × 10^4^ cells) were dissociated and resuspended in 2 μL PBS. Mice were anesthetized and placed in a stereotactic frame. A hole was made at 0.5 mm to the anterior and 2.0 mm to the right of the bregma. The ALTS1C1 cells (2 μL) were injected using a 10 μL Hamilton syringe with a 26S-gauge needle mounted in a stereotactic holder. The syringe was lowered to a depth of 3.0 mm. Cells were injected at a rate of 0.4 μL/min. The syringe was withdrawn slowly to a depth of 1.0 mm for a duration of 5 min to prevent reflux after injection. The skull was cleaned and the incision sutured. The survival of each mouse was recorded.

### 2.13. Statistical Analysis

The data are presented as means ± the standard error of the mean (SEM). The statistical analysis between the two samples was compared using a Student’s *t*-test. * *p*-Values < 0.05 are considered significant and are indicated in the figure legends. All statistical analyses were performed using SigmaPlot software (version 10.0, Systat Software Inc., San Jose, CA, USA).

## 3. Results

### 3.1. B1R OE Promotes GBM Cell Migration

**Figure 1:**
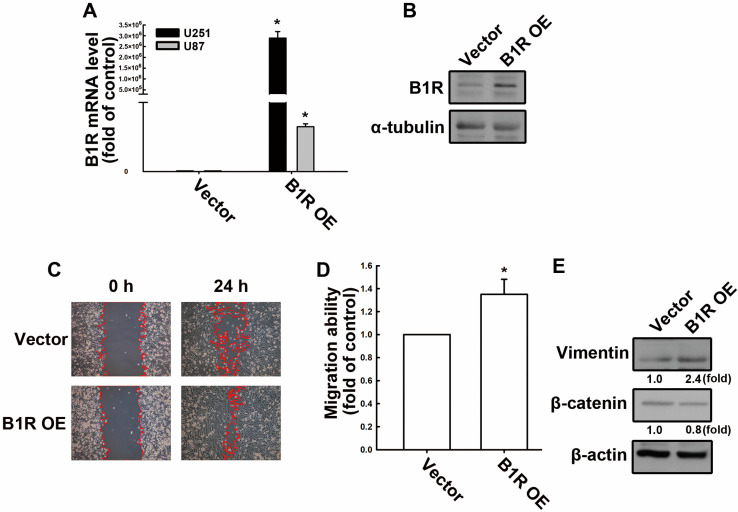
B1R OE enhances the migration ability of human GBM cells. (**A**) U251 and U87 human GBM cells were transfected with an EV or B1R wild-type (B1R-OE) plasmid for 24 h. The B1R mRNA levels were quantified using real-time polymerase chain reaction (PCR). (**B**) U251 human GBM cells were transfected with B1R-overexpressing cells for 24 h. The expression of the B1R protein was determined using Western blotting. U251 cells were transfected with an EV or a B1R-OE plasmid for 24 h. The migration ability was determined using a wound healing assay (100x magnification) (**C**). The quantitative results are shown in (**D**). The expression levels of vimentin and β-catenin were determined using Western blotting (**E**). The results are representative of at least three independent experiments and are presented as the mean ± standard error of the mean (SEM). The results were analyzed using the Student’s *t*-test. * *p* < 0.05 compared with the vector group.

We developed a B1R OE model to elucidate the role of B1R in GBM progression. First, we constructed B1R-overexpressed plasmid DNA. Subsequently, GBM cells were transfected with this B1R-overexpressing plasmid (hereafter referred to as B1R-OE GBM cells) or an empty vector (EV; hereafter referred to as EV GBM cells). We observed that the B1R-OE GBM cells exhibited significantly higher B1R mRNA (Figure 1A) and protein (Figure 1B) expression levels than the EV GBM cells. Thus, this confirmed our B1R OE model, which was used for the subsequent analyses. We next determined whether B1R OE affected the GBM cell motility. The results reveal that B1R OE intensified the rate of GBM cell migration (Figure 1C). The migration ability of the B1R-OE GBM cells was significantly higher than that of the EV GBM cells (Figure 1D). Furthermore, we examined changes in the expression of the epithelial–mesenchymal transition (EMT) markers, which are highly associated with cancer cell motility and progression [37]. We found that the expression of vimentin was higher in the B1R-OE GBM cells than in the EV GBM cells (Figure 1E). By contrast, the β-catenin expression decreased in the B1R-OE GBM cells (Figure 1E). These results suggest that B1R expression in GBM promotes tumor motility.

### 3.2. B1R OE in GBM Cells Induces the Expression of Adhesion Molecules and Immune Checkpoints

**Figure 2:**
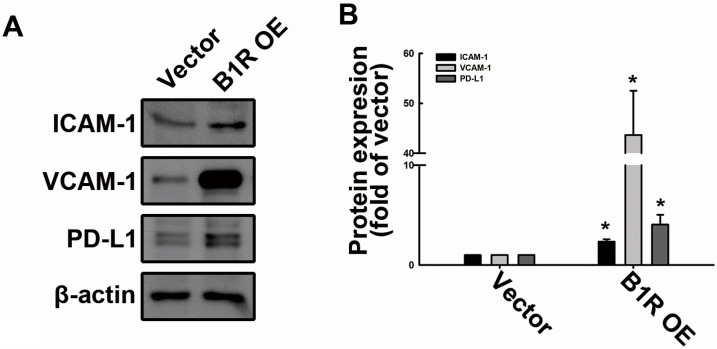

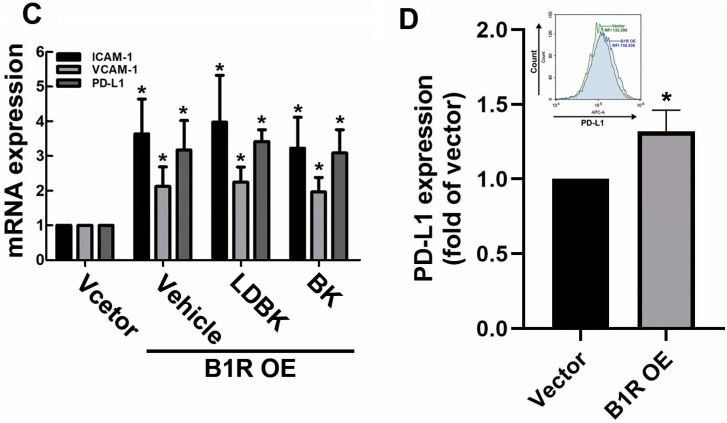
B1R OE promotes the expression of adhesion molecules and immune checkpoints. U251 human GBM cells were transfected with an EV or a B1R-OE plasmid for 24 h. (**A**) The protein expression levels of ICAM-1, VCAM-1, and PD-L1 were determined using Western blotting. The quantitative results are shown in (**B**). (**C**) After transfection with the EV or B1R-OE plasmid for 24 h, the cells were treated with LDBK (100 nM) or bradykinin (1 μM) for another 24 h, and the mRNA expression levels of ICAM-1, VCAM-1, and PD-L1 were quantified using real-time PCR. (**D**) The surface expression of PD-L1 was determined using flow cytometry. The results are representative of at least three independent experiments and are presented as the mean ± SEM. The results were analyzed using the Student’s *t*-test. * *p* < 0.05 compared with the vector group.

Increasing evidence indicates that CAMs regulate tumor cell invasion, migration, and proliferation [38]. Our previous study and other research groups have reported that ICAM-1 and VCAM-1 upregulation could mediate monocyte and macrophage binding to GBM cells and could promote GBM progression [29,39]. In the present study, we examined whether B1R OE in GBM cells could regulate the production of adhesion molecules and immune checkpoints. Accordingly, we transfected human GBM cells with the B1R-overexpressed plasmid for 24 h. We then determined the protein expression levels of the adhesion molecules ICAM-1 and VCAM-1 and the immune checkpoint programmed death (PD)-L1 in the cells. The results revealed that the protein expression levels of ICAM-1 and VCAM-1 were significantly higher in the B1R-OE GBM cells than in the EV GBM cells (Figure 2A,B). Notably, the expression of PD-L1 was also upregulated in the B1R-OE GBM cells (Figure 2A,B). The mRNA expression levels of ICAM-1, VCAM-1, and PD-L1 were also significantly higher in the B1R-OE GBM cells than in the EV GBM cells (Figure 2C). Notably, treatment with the B1R ligand Lys-des(Arg(9))-bradykinin (LDBK) or bradykinin failed to increase the expression of ICAM-1, VCAM-1, or PD-L1 in the B1R-OE GBM cells (Figure 2C). We further examined the surface protein expression of PD-L1 in the GBM cells using flow cytometry. Our results reveal an increased surface protein expression of PD-L1 in the B1R-OE GBM cells (Figure 2D). These findings demonstrate that B1R regulates the production of adhesion molecules and PD-L1 in GBM cells. 

### 3.3. B1R OE Induces Monocyte Binding to GBM Cells

**Figure 3:**
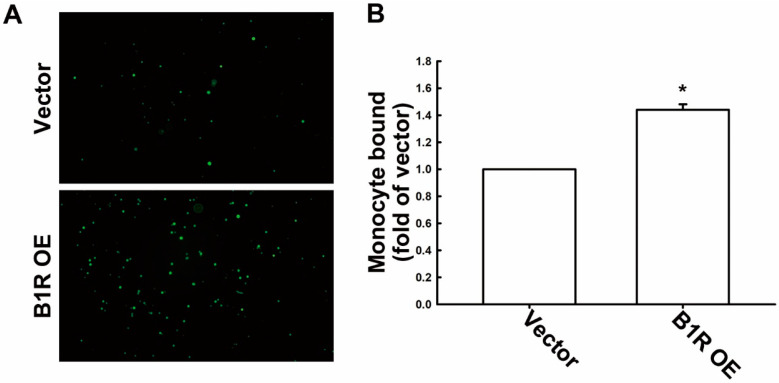
B1R regulates monocyte binding to GBM cells. (**A**) U251 human GBM cells were transfected with an EV or a B1R-OE plasmid for 24 h. Human monocytes (THP-1 cells) labeled with the florescent dye BCECF-AM (green dots) were added to the GBM cells for 30 min. After several washes, THP-1 bound to GBM cells, which was observed under a fluorescence microscope (100x magnification). The quantitative results are shown in (**B**). The results in the bar graph are presented as the mean ± SEM (*n* = 3). The results were analyzed using the Student’s *t*-test. * *p* < 0.05 compared with the vector group.

Accumulating evidence demonstrates that macrophages play a key role in the TME and are associated with tumor angiogenesis, invasion, and metastasis [40]. During cancer progression, monocytes are actively recruited and attached to the tumor sites, where they differentiate into macrophages that contribute to tumor progression [41]. In the present study, we observed that B1R OE upregulated ICAM-1 and VCAM-1, which were previously reported to facilitate monocyte binding to GBM cells. Accordingly, we investigated the effect of B1R OE on monocyte binding to GBM cells. The results indicate that B1R OE engendered enhanced monocyte binding to the B1R-OE GBM cells relative to that of the EV GBM cells (Figure 3A). Figure 3B illustrates the quantitative analysis results regarding monocyte binding to GBM cells.

### 3.4. B1R OE Inhibits ROS Production in GBM Cells

**Figure 4:**
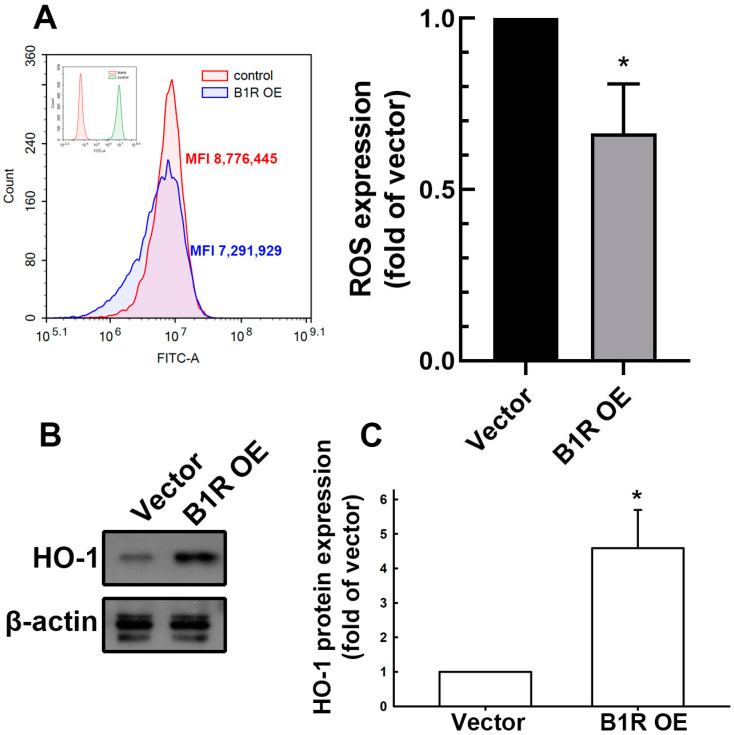
B1R OE leads to reduced ROS production and increased HO-1 expression. (**A**) U251 human GBM cells were transfected with an EV or a B1R-OE plasmid for 24 h. After 40 min of incubation with 2′,7′-dichlorodihydrofluorescein diacetate, ROS production was detected using flow cytometry. The quantitative results are shown in the right panel. (**B**) The expression of HO-1 was determined using Western blotting. The quantitative results are presented in (**C**) as the mean ± SEM (*n* = 3). The results were analyzed using the Student’s *t*-test. * *p* < 0.05 compared with the vector group.

In general, ROS tightly regulate cellular stability, including proliferation, survival, and apoptosis, through several signaling cascades. In cancer, excessive ROS production may damage DNA directly by promoting cellular mutation [42]. Using flow cytometry, we investigated whether B1R OE affects intracellular ROS production in GBM cells. The analysis results reveal that ROS production in the B1R-OE GBM cells was considerably lower than that in the EV GBM cells (Figure 4A). Furthermore, we examined the expression of heme oxygenase-1 (HO-1), an endogenous antioxidant protein, in the cells after B1R alteration. Our results indicate that the expression of HO-1 was markedly higher in the B1R-OE GBM cells than in the EV GBM cells (Figure 4B,C). These findings suggest that B1R promotes GBM progression partly by influencing the free radical balance of GBM cells, thus providing a favorable environment for tumor cells. 

### 3.5. B1R OE Induces Expression of Protumorigenic Cytokines and Chemokines in GBM Cells

**Figure 5:**
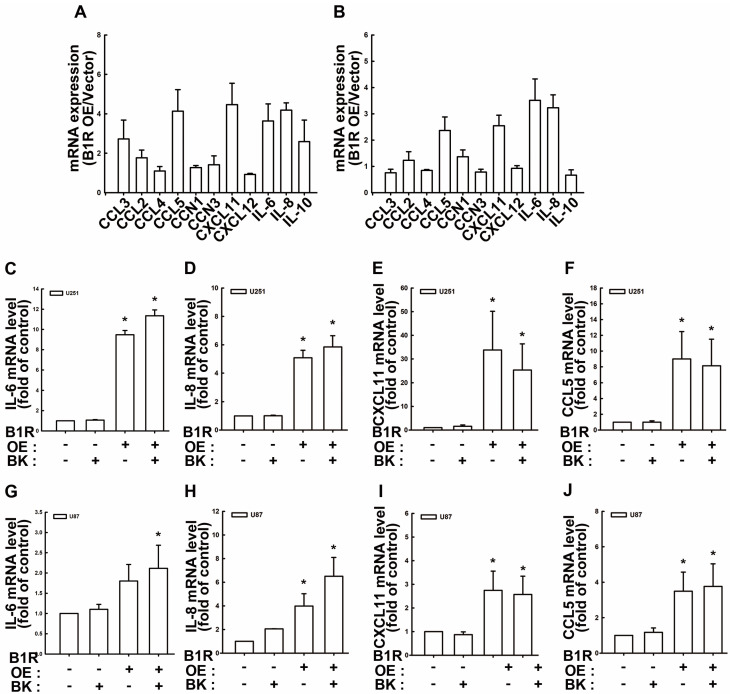
B1R OE promotes protumorigenic cytokine and chemokine expression. (**A**) U251 and (**B**) U87 human GBM cells were transfected with an EV or a B1R-OE plasmid for 24 h. The mRNA expression levels of chemokines and cytokines were determined using real-time PCR. The quantitative results were normalized using the EV-transfected GBM cells. (**C**–**F**) U251 and (**G**–**J**) U87 human GBM cells were transfected with an EV or B1R-OE plasmid for 24 h before treatment with bradykinin (1 μM) for an additional 24 h. The expression levels of (**C**,**G**) IL-6, (**D**,**H**) IL-8, (**E**,**I**) CXCL11, and (**F**,**J**) CCL5 were determined through real-time PCR. Data in the bar graph are presented as the mean ± SEM (*n* = 3). The results were analyzed using the Student’s *t*-test. * *p* < 0.05 compared with the vector group.

Soluble mediators in the TME, such as cytokines and chemokines, considerably affect the immune regulation and recruitment of TAMs, resulting in ineffective antitumor responses and contributing to tumor progression [43]. We demonstrated that B1R negatively mediated ROS production in GBM cells, consequently leading to unfavorable anticancer responses. Accordingly, we examined the effect of B1R OE on the expression of cytokines and chemokines. The results revealed that B1R OE promoted IL-6, IL-8, CXCL11, and CCL5 expression in both U251 and U87 human GBM cells (Figure 5A,B). Moreover, we found that treatment with the bradykinin ligand failed to improve IL-6, IL-8, CXCL11, or CCL5 production in B1R-OE U251 cells (Figure 5C–F). In addition, treatment with the bradykinin ligand did not affect the upregulation of IL-6, IL-8, CXCL11, or CCL5 in B1R-OEU87 cells (Figure 5G–J). Similarly, treatment with LDBK did not influence the enhancement of protumorigenic cytokines and chemokines in B1R-OE GBM cells (Supplementary Materials Figure S1). 

### 3.6. B1R OE in GBM Cells Affects Macrophage Polarization and PD-L1 Expression in Macrophages

**Figure 6:**
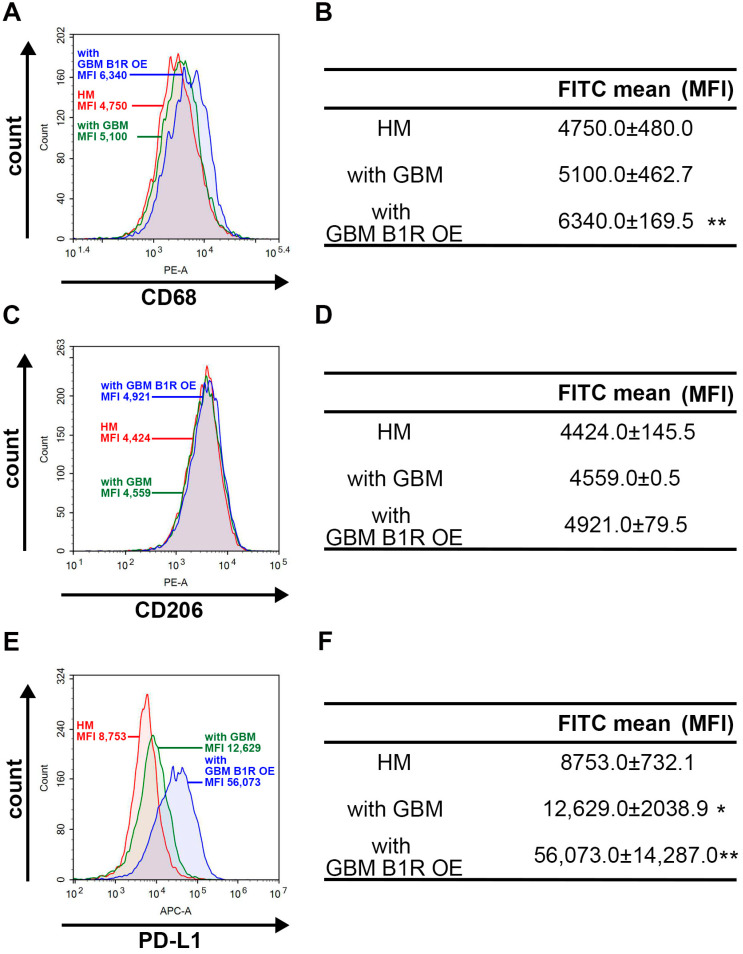
B1R OE in GBM modulates macrophage polarization and PD-L1 expression in TAMs. Green fluorescent protein (GFP)-expressing GBM cells were transfected with an EV or a B1R-OE plasmid for 24 h and cocultured with human THP-1-differentiated macrophage for another 24 h. The surface expression levels of (**A**) CD68, (**C**) CD206, and (**E**) PD-L1 in the human macrophage were determined using flow cytometry. The quantitative results for (**B**) CD68, (**D**) CD206, and (**F**) PD-L1 were determined. The units of measurement for the FITC is mean fluorescence intensity (MFI). The results were analyzed using the Student’s *t*-test. * *p* < 0.05 compared with the HM group. ** *p* < 0.05 compared with the parental GBM group.

A previous study demonstrated that GBM tumors extensively infiltrated immunosuppressive CD68^+^ macrophage populations, which were coexpressed with PD-L1, and that low expression levels of CD3^+^ T-cell populations compromised GBM prognosis [44]. To examine the effects of B1R on immune modulation in GBM cells, the present study developed a coculture system comprising human macrophages (HMs) and B1R-OE GBM cells (hereafter referred to as HM–BIR-OE GBM system) or HMs and EV GBM cells (hereafter referred to as HM–EV GBM system). We observed that the expression of CD68, an M1 macrophage marker, was higher in the HM–BIR-OE GBM system than in the HM–EV GBM system (Figure 6A,B). However, B1R OE did not influence the expression of CD206, an M2 macrophage marker (Figure 6C,D). We also observed that the expression of PD-L1 was upregulated in the HM–BIR-OE GBM system (Figure 6E,F). Notably, the expression of PD-L1 was higher in the HM–BIR-OE GBM system than in the HM–EV GBM system (Figure 6E,F). Overall, our findings demonstrate that B1R OE in GBM cells polarizes the surrounding macrophages into M1 phenotypes and promotes the expression of PD-L1, which are responsible for the immunosuppressive response in TME. 

### 3.7. B1R OE in GBM Reduces the Survival Rate of an Intracranial Xenograft Mouse Model 

**Figure 7:**
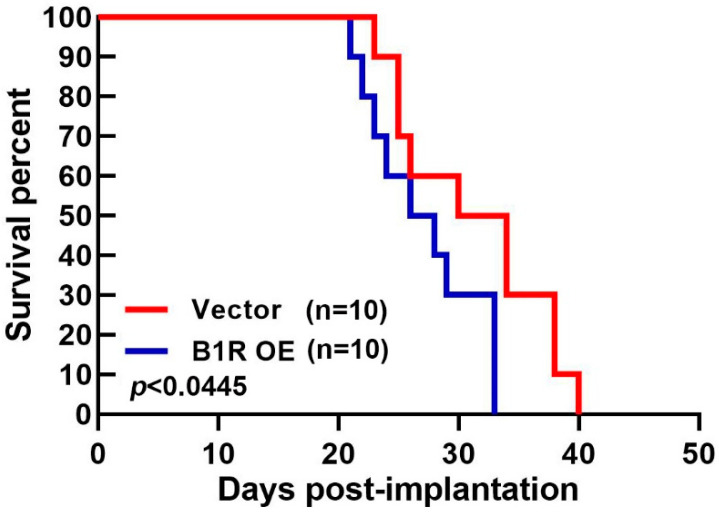
B1R OE reduces survival rates in an intracranial xenograft mouse model. EV-transfected or B1R-overexpressing ALTS1C1 cells were implanted into the brains of mice. The survival rates of the mice after transplantation were recorded, as indicated by the Kaplan–Meier survival curve. The results were analyzed using survival analyses. *p* < 0.05 compared with the vector group.

To verify the influence of B1R on GBM progression, we explored the effect of B1R OE in a GBM-bearing mouse model. We implanted B1R-overexpressing ALTSC1 GBM cells or EV-transfected GBM cells into the brains of mice and then recorded their survival rate (in days). The survival rate of the mice implanted with the B1R-OE GBM cells was lower than that of the mice implanted with the EV-transfected GBM cells (Figure 7). These findings suggest that B1R OE in GBM contributes to GBM progression and reduces survival rates.

## 4. Discussion

In cancer cells, high ROS levels may engender oxidative stress, and this stress may be due to increased metabolic activity, mitochondrial dysfunction, increased cellular receptor signaling, or immune cell infiltration [45]. ROS can mediate the growth, proliferation, metabolism, and survival of cancer cells [46]. Additionally, ROS are associated with numerous tumor suppressor genes that control cell cycle arrest, DNA damage repair, cell migration and differentiation, and programmed cell death [47]. A previous study reported that ROS induced apoptosis in GBM cells through the mitogen-activated protein kinase pathway [48]. Another study noted that treatment with a pharmacological agent led to anti-GBM activity by stimulating endogenous ROS production, which resulted in the programmed cell death of GBM cells [49]. In addition, a recent study revealed that bradykinin promoted the intracellular production of superoxide and nitric oxide, which further supported melanoma cell invasion and migration [50]. HO-1, an antioxidant protein, is expressed in many cancers and can promote tumor progression through multiple mechanisms [51]. Several studies have determined that HO-1 offers potent proangiogenic properties, in addition to its excellent anti-inflammatory and antioxidant effects [52]. Furthermore, HO-1 regulates inflammatory responses and mediates immunosuppression in tumor cells [53]. HO-1 can also prevent apoptosis and autophagy in tumor cells [54], and it is involved in a series of complex molecular mechanisms that drive cell proliferation, survival, and metastasis in GBM [55,56]. Yang et al. identified the regulatory role of bradykinin in neuronal apoptosis [57]. Specifically, they reported that B2R upregulates HO-1 expression and activity, contributing to neuronal apoptosis [57]. In the present study, we demonstrated that B1R promotes HO-1 and facilitates immune escape of GBM cells from oxidative stress-induced cell death, thereby promoting the development of GBM tumors.

PD-L1, a ligand of PD-1, is an immune checkpoint inhibitor that suppresses immune functions [58]. PD-L1 plays a key role in the immune escape of tumors, regulates tumor-intrinsic events, and promotes tumor progression [59]. Furthermore, PD-L1 promotes the EMT in cancer cells by inhibiting EMT transcription factor disruption [60]. PD-L1 OE in GBM can evade immune surveillance [61,62]. After chemotherapeutic drug treatment, the upregulation of PD-L1 expression contributes to the immune escape of GBM cells [63]. An increasing body of evidence indicates that PD-L1 is highly expressed in gliomas [64] and positively correlated with the clinical grade of gliomas [65]. Specifically, studies have demonstrated that PD-L1 was detected in approximately 90% of newly diagnosed GBM cases and that PD-L1 was detected in nearly 70% of recurrent GBM cases [66,67]. Higher PD-L1 expression is usually correlated with poorer outcomes in patients with glioma [68,69]. Our previous study revealed that high PD-L1 expression levels in protumoral TAMs in GBM were positively correlated with GBM progression [70]. Additionally, PD-L1 activates the PD-1 receptor on T cells and suppresses the immune activity of T cells [71]. PD-L1 expression was also reported to be negatively correlated with CD8^+^ T cells [72]. PD-L1 influences macrophage polarization by promoting the switching of macrophages toward an M2 phenotype [73]. Anti-PD-L1 treatment alters the phenotypes of tumor macrophages and enhances T-cell activities [74]. In GBM, PD-L1 recruits M2 macrophages and contributes to immunosuppression [72]. Moreover, the expression of PD-L1 on infiltrating lymphocytes indicates that specific immunosuppressive pathways may be involved in GBM [67]. The present study demonstrated PD-L1 upregulation in B1R-OE GBM cells in addition to revealing that that B1R OE significantly upregulated PD-L1 in TAMs in GBM. 

CAMs, including ICAM-1 and VCAM1, are involved in the interaction and recruitment of immune cells to tumor sites [39]. B1R was found to promote the adhesion of leukocytes to endothelial cells through the induction of ICAM-1 [75]. Moreover, treatment with B1R agonists could attenuate melanoma metastasis by inhibiting VCAM-1 expression, indicating the regulatory role of bradykinin and adhesion molecules in the host immune response of tumor cells [76]. Our previous study demonstrated that B1R increased the production of IL-8, which contributed to the migration of GBM cells by regulating STAT3 and SP-1 [77]. We also found that B1R induced cyclooxygenase-2 expression and stimulated GBM cell migration [78]. To clearly understand the regulatory roles of B1R in GBM progression, the present study transfected B1R wild-type plasmid DNA into glioma cell lines to overexpress B1R; this study subsequently investigated the effect of B1R OE on GBM and immune-related cells. Consistent with other studies, our study revealed that B1R OE enhanced the migration of GBM cells and the expression of vimentin, an EMT marker. Moreover, B1R OE induced ICAM-1 and VCAM-1 production, which further enhanced THP-1 monocyte binding to GBM cells.

Chemokines are chemotactic and structurally related cytokines and can be divided into the following subfamilies according to the location of the first two N-terminal cysteine residues: CC, CXC, CX3C, and XC [79]. Chemokines control leukocyte infiltration to the tumor area through the activation of G-protein-coupled receptors, thus regulating the host immune response to cancer cells [80]. CCL5 is expressed by various cancer cells and plays different roles in shaping TMEs [81]. Huang et al. reported that CCL5 promoted the cell surface expression of αvβ3 integrin and facilitated the migration and invasion of human lung cancer cells [82]. In particular, CCL5 contributes to immune escape in colorectal cancer by boosting the toxicity of regulatory T cells against cytotoxic T cells, leading to the cell apoptosis of cytotoxic T cells in tumors [83]. A previous study revealed elevated mRNA and protein expression of CCL5 in tumor tissue samples collected from patients with high-grade glioma [84]. In addition to CCL5 expression, high CXCL11 expression levels were noted to be associated with a poorer prognosis in most cancers [85]. For example, a study noted that CXCL11 enhanced cancer proliferation and metastasis in pancreatic cancer [86]. Furthermore, IL-6 and IL-8 are major cytokines in the TME that regulate multiple signaling pathways, including apoptosis, survival, proliferation, angiogenesis, invasiveness, migration, and metastasis, in various malignant tumors [87,88,89]. Studies have observed that IL-6 expression in patients with GBM accelerated tumor progression and affected patient prognosis [90,91]. Moreover, a study demonstrated that bradykinin mediated the migration and invasion of colorectal cancer by inducing IL-6 production [88]. IL-8 is produced by tumor cells themselves or released by immune cells, and it provides a favorable environment for tumor proliferation, migration, and invasion [89,92]. B1R also modulates macrophage accumulation and chemokine expression [93]. Our previous study showed that the IL-8 expression level was directly correlated with the B1R expression level in a GBM model [77]. We demonstrated that the level of IL-8 expression in U87 human GBM cells, which exhibited greater B1R expression, was significantly higher than that in GBM8901 cells, which exhibited lower B1R expression; thus, bradykinin plays a key role in the stimulation of IL-8 in GBM cells [77]. The results of the present study reveal that B1R upregulation promoted the mRNA expression of IL-6 and IL-8 in GBM cells, supporting our previous study’s findings. We also determined the effect of B1R on the production of CXCL11 and CCL5. In addition, the present study found that administering bradykinin treatment to B1R-overexpressing cells did not improve the production of such cytokines or chemokines. 

Chemokines are closely related to various immune cells in the TME. For example, research has reported that chemokine deficiency was associated with enhanced intratumoral infiltration of cytotoxic CD8^+^ T cells [94] and that it promoted tumor immune escape [83]. Chemokines were also revealed to participate in the recruitment of TAMs [95]. Studies have also reported that TAM infiltration promoted cancer progression and that it was correlated with a poor prognosis in numerous cancers [96] and with treatment resistance [97]. TAMs secrete chemokines and activate the PI3K–Akt–mTOR signaling pathways, increasing endocrine resistance in breast cancer [98]. GBM has a highly inflammatory TME in which microglia and macrophages infiltrate approximately 30% of the tumor mass [99]. Furthermore, chemokines can affect TAM polarization [100]. The present study found that the expression of CD68, an M1 macrophage surface marker, increased in the HM–BIR-OE GBM system, demonstrating the regulatory roles of B1R in macrophage polarization in the TME. 

## 5. Conclusions

This study demonstrated that B1R in GBM regulates the TME and aggravates GBM progression. Our results show that B1R OE stimulated the expression of ICAM-1 and VCAM-1—adhesion molecules—thus promoting the migration and attachment of myeloid cells, particularly monocytes, to tumor cells. Moreover, B1R OE induced the production of protumorigenic cytokines and chemokines, namely, IL-6, IL-8, CXCL11, and CCL5, which regulated the immune response and provided a favorable environment for GBM progression. B1R notably stimulated the expression of the endogenous antioxidant HO-1 in GBM cells, which consequently suppressed the intracellular production of ROS, leading to protective effects against oxidative stress. We also found that B1R OE in GBM cells promoted the polarization of TAMs toward the M1 phenotype. Furthermore, B1R induced the expression of the immune checkpoint PD-L1 in GBM cells and macrophages, enabling them to escape attacks from immune cells. Finally, B1R OE promoted tumor growth and reduced survival rates in vivo. Our findings can be used as a reference to develop novel therapeutic strategies for GBM treatment. 

## Data Availability

Data are contained within the article and the Supplementary Materials.

## Supplementary Materials

The following supporting information can be downloaded at: https://www.mdpi.com/article/10.3390/antiox12081533/s1, Figure S1: Effects of LDBK on B1R OE GBM cells.
