# Supplementary material for: Bradykinin B1 Receptor Affects Tumor-Associated Macrophage Activity and Glioblastoma Progression
Source: Antioxidants (Basel). 2023 Jul 31;12(8):1533. doi: 10.3390/antiox12081533 (PMC10451655; doi:10.3390/antiox12081533)
Supplement: Supplementary file 1 [file antioxidants-12-01533-s001.zip › antioxidants-2485655-supplementary.pdf]

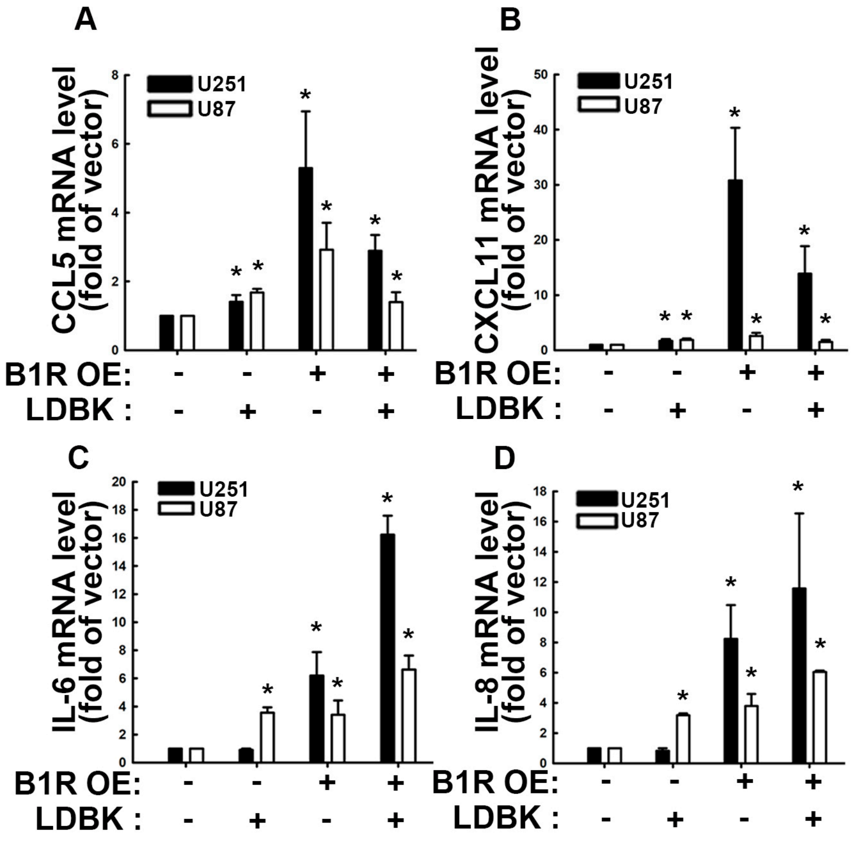

Figure S1. Effects of LDBK on B1R OE GBM cells. U251 and U87 human GBM cells were transfected with an EV or B1R-OE plasmid for 24 h before treatment with LDBK (100 nM) for an additional 24 h. The expression levels of CCL5 (A), CXCL11 (B), IL-6 (C), and IL-8 (D) were determined through real-time PCR. Data in the bar graph are presented as the mean ± SEM (n = 3). The results were analyzed using Student's t-test. *$p < 0.05$ compared with the vector group.
